# Difficult management of pancreatic pseudocyst drainage in a 4-year-old child

**DOI:** 10.1055/a-2299-2253

**Published:** 2024-04-24

**Authors:** Jingqing Zeng, Zhaohui Deng, Sheng Ding

**Affiliations:** 1 Gastroenterology, Shanghai Childrenʼs Medical Center, Shanghai Jiatong University, Shanghai, China

**Fig. 1:**
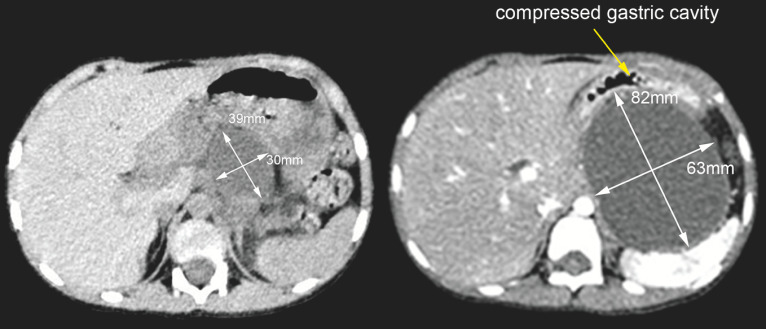
Computed tomography (CT) showed an enlarged pancreatic pseudocyst at 6-month follow-up after stent drainage of the pancreatic duct in a 4-year-old child.

A 4-year-old boy with acute lymphoblastic leukemia had recurrent pancreatitis with pancreatic pseudocyst formation following asparaginase chemotherapy. At 6 months after stent drainage of the pancreatic duct, computed tomography (CT) revealed an enlarged pancreatic pseudocyst ( Fig. 1 ).

However, the massive pseudocyst compressed the gastric lumen, which was relatively small due to the patient’s young age, which led to difficulty in selecting a puncture site. Finally, the junction of the esophagus and cardia was selected for puncture. The puncture tract was expanded using an 8-mm dilation catheter. The intention was that, for drainage, two double-pigtail stents (7 Fr, 7 cm), were to be deployed from the esophagus, with their ends eventually coiling in the gastric lumen at the cardia.

However as the first stent was being placed, straightforward release of the pigtail end was precluded by the small space available. When its guidewire was withdrawn, the entire stent slid into the pseudocyst because of esophageal compression and inertial coiling of the stent.

Removal of the stent from the pseudocyst lumen was challenging. The puncture site was identified using a gastroscope with a transparent cap (Olympus; diameter 9.2 mm). A foreign body forceps was advanced through the dilated puncture channel into the lumen of the pseudocyst, under fluoroscopic guidance and along the second of the guidewires that had been inserted for stent placement. The stent was successfully removed and replaced by a 7-Fr curved transnasal pancreaticobiliary drain ( Video 1 ).

Challenging management of pancreatic pseudocyst drainage in a 4-year-old child.Video 1

**Fig. 2:**
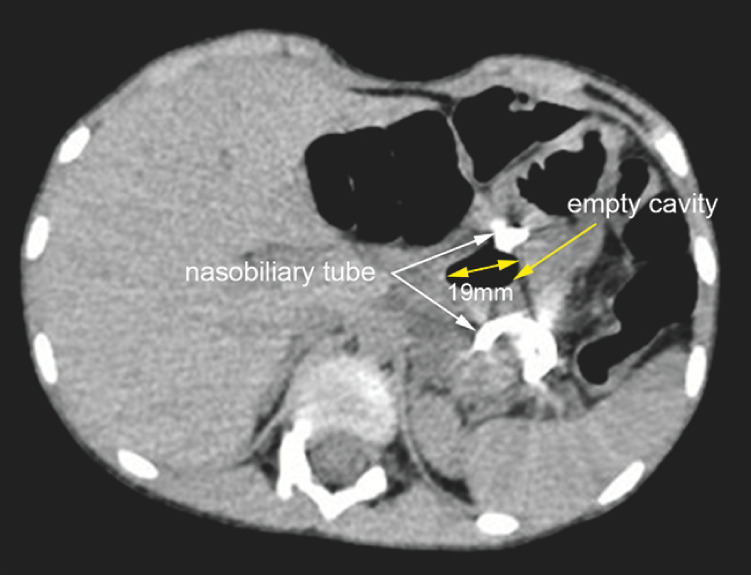
CT at 1 week after start of external nasobiliary drainage showed shrinking of the pseudocyst to 20.9 × 16.3 × 21.6 mm.

**Fig. 3:**
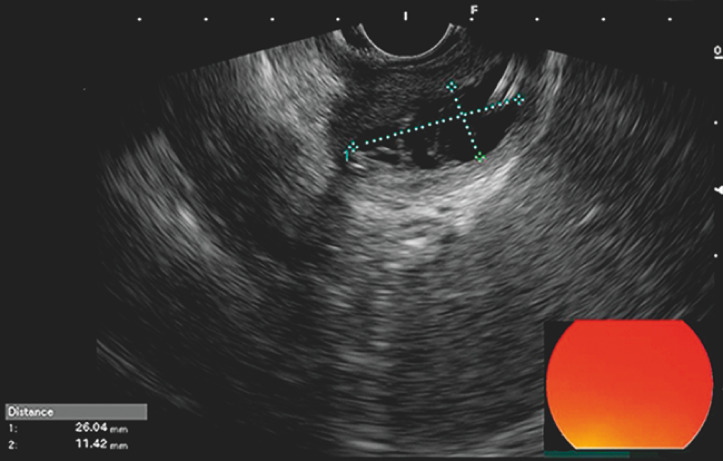
Endoscopic ultrasound after 2 weeks of drainage showed shrinking of the pseudocyst to approximately 26 × 11 mm.

On CT re-examination at 1 week post-procedure, the pseudocyst was markedly smaller ( Fig. 2 ; 20.9 × 16.3 × 21.6 mm). On endoscopic ultrasonography at 2 weeks post-procedure, the lumen was completely drained of cystic fluid and presented as an empty cavity; the cyst was 26 × 11 mm ( Fig. 3 ). Subsequently, the transnasal pancreaticobiliary drain was removed, no complications (e.g., esophagopleural fistula) were observed on postoperative follow-up, and pancreatic enzymes returned to normal levels.

The small lumen of the digestive tract in children is a major challenge in endoscopic treatment. Puncture and drainage at the gastroesophageal junction are feasible in special cases. In this case successful drainage was achieved at this site, albeit suggesting that external drainage may be more appropriate for a puncture site near the esophagus.

Endoscopy_UCTN_Code_CPL_1AL_2AD

